# Can Integrin Agonists Have Cards to Play against Cancer? A Literature Survey of Small Molecules Integrin Activators

**DOI:** 10.3390/cancers9070078

**Published:** 2017-07-05

**Authors:** Alessandra Tolomelli, Paola Galletti, Monica Baiula, Daria Giacomini

**Affiliations:** 1 Department of Chemistry “G. Ciamician”, University of Bologna, Via Selmi 2, 40126 Bologna, Italy; alessandra.tolomelli@unibo.it (A.T.); paola.galletti@unibo.it (P.G.); 2 Department of Pharmacy and Biotechnology, University of Bologna, Via Irnerio 48, 40126 Bologna, Italy; monica.baiula@unibo.it

**Keywords:** integrins, agonists, ligands, signaling, lactams, RGD mimetics, activation

## Abstract

The ability of integrins to activate and integrate intracellular communication illustrates the potential of these receptors to serve as functional distribution hubs in a bi-directional signal transfer outside-in and inside-out of the cells. Tight regulation of the integrin signaling is paramount for normal physiological functions such as migration, proliferation, and differentiation, and misregulated integrin activity could be associated with several pathological conditions. Because of the important roles of integrins and their ligands in biological development, immune responses, leukocyte traffic, haemostasis, and cancer, their potential as therapeutic tools is now widely recognized. Nowadays extensive efforts have been made to discover and develop small molecule ligands as integrin antagonists, whereas less attention has been payed to agonists. In recent years, it has been recognized that integrin agonists could open up novel opportunities for therapeutics, which gain benefits to increase rather than decrease integrin-dependent adhesion and transductional events. For instance, a significant factor in chemo-resistance in melanoma is a loss of integrin-mediated adhesion; in this case, stimulation of integrin signaling by agonists significantly improved the response to chemotherapy. In this review, we overview results about small molecules which revealed an activating action on some integrins, especially those involved in cancer, and examine from a medicinal chemistry point of view, their structure and behavior.

## 1. Introduction

**Figure 1:**
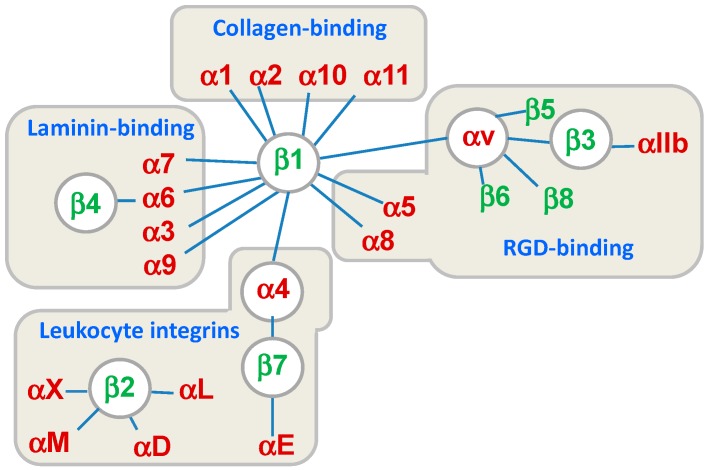
Classification of the 24 integrin heterodimers according to the combination of the subunits α and β, their specific ligands, or cell type. RGD: Arg-Gly-Asp.

Integrins are cell surface receptors structurally comprised of non-covalent associations between α and β subunits. Both subunits are type I transmembrane glycoproteins that contain a relatively large extracellular domain, a single transmembrane domain, and a short cytoplasmic tail [1,2]. In mammals, 18 α subunits and eight β subunits are non-covalently associated to form 24 different integrin αβ heterodimers expressed differently in particular tissues. In addition to this tissue specificity, each integrin exhibits a distinct binding affinity to particular ligands (Figure 1) [3,4]. The α-subunits have the greatest influence on ligand-binding specificity, and define different integrin families with specificity for Arg-Gly-Asp (RGD) motifs (α_IIb_, α_V_, α_5_, and α_8_), intercellular adhesion molecules and inflammatory ligands (α_4_, α_L_, α_M_, α_X_, and α_D_), collagens (α_1_, α_2_, α_10_, and α_11_), and laminins (α_3_, α_6_, and α_7_). 

Integrins are not just adhesion receptors that mediate dynamic adhesive cell-cell and cell-matrix interactions, but they can transmit information on the chemical identity and physical state of their ligands into cells, to regulate cell migration, cell survival, and growth. The activation of intracellular signaling pathways controls cell shape, motility, proliferation, survival, and cell-type-specific gene expression [2]. Adhesion signaling via integrins is, therefore, a key contributor to both health and disease [5,6,7].

Integrins are normally inactive with low affinity for their endogenous ligands, but they undergo rapid activation upon various stimuli [8,9]. To convert integrins into active states with different ligand binding affinities, intracellular signaling (inside-out signaling) is required. As bidirectional receptors, integrins can also transmit signals back into cells: upon ligand binding in the extracellular domain, they transmit an outside-in signaling that regulates cell shape, migration, growth, and differentiation. 

The ability of integrins to bind and associate with various components of the extracellular matrix (ECM) or soluble ligands largely depends on the structural conformations of the two subunits α and β, and distinct conformations are crucial for regulating both inside-out and outside-in cell signaling [4,10,11]. 

**Figure 2:**
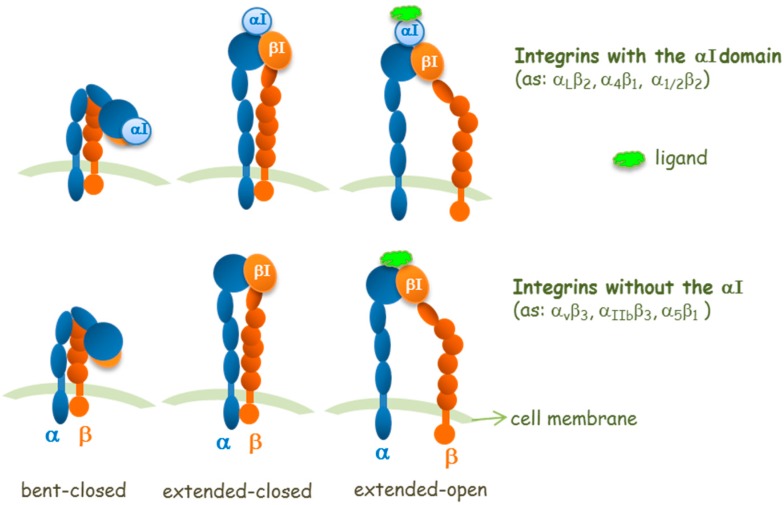
Schemes for three conformational states of αI-integrins and integrins without the αI domain, and interaction sites of a ligand.

Concerning the integrin site for ligand binding and from a structural point of view, it is important to distinguish the two types of α-subunits in integrins: those with or those without an inserted domain, named the αI domain (Figure 2). In αI-integrins, the αI domain is the binding site for ligands, whereas in integrins without the αI domain, the ligand-binding site is formed at the interface between the α-subunit and the β-subunit in its βI domain [11]. Thus, in integrins without an αI domain, the β subunit contributes to modulate the ligand specificity. The αI and βI domains are structurally homologous and contain metal ion-dependent adhesion sites (MIDAS) which are able to bind Asp, Glu, or carboxylic acid residues in ligands. In RGD-binding integrins (without αI), the Arg of RGD binds the α-subunit while the Asp of RGD coordinates to the Mg^2+^ ion in the β subunit βI domain MIDAS. The elucidation of this mode of ligand binding explains the cation dependence of ligand binding and cell adhesion.

Structural studies (crystallography, nuclear magnetic resonance, NMR, and electron microscopy studies) have revealed three overall conformational states: a bent, an extended-closed, and an extended-open conformations (Figure 2); these may correspond to a low affinity conformer, an activated, and an activated together with ligand-occupied integrin conformers, respectively [12]. 

However, in some circumstances, the bent form can engage ligands such as fibronectin fragments [13] or a small molecule that is not a RGD mimetic [14], which do not prime the receptor. The concept that the extended open integrin conformation corresponds to the conformation with high affinity for the ligand is well accepted for some integrins, whereas for others there are controversial aspects [15]. Binding of extracellular ligands also stabilizes the extended open conformation, which shows enhanced separation of the integrin intracellular tails that, in turn, transmits signals to the cytoplasm (outside-in signaling). Because the equilibria of conformational changes and ligand binding are thermodynamically linked [16], it is reasonable to assume that the extended closed conformer could have an intermediate affinity for ligands [17].

Regulation of integrin affinity by ligands should be viewed as a shifting of the dynamic equilibrium between closed, intermediate and open conformers [18]. For many, if not all integrins [19], such conformational changes (“activation”) are required to actuate their adhesive function and signal transduction [19,20]. 

Integrins, like most other cell surface receptors, are heavily glycosylated. The significance of the great variation in number of glycosylation sites among integrin subunits is currently unknown. Recently, it was reported that *N*-glycans affect the conformational equilibria of integrins and their activation [21]. Moreover, a decrease in the number of *N*-glycosylation sites on integrin α_5_β_1_ stabilizes its bent-closed and extended-closed conformations and lowers the ligand binding affinity [22].

Integrins form part of a multidimensional system in which complex cellular signaling might be influenced by functional cross-talk between the membrane receptors, such as integrins, growth factors receptors, cadherins, matrix metalloproteinases (MMPs), etc. It was demonstrated that these cross-talk interactions are very important for cell proliferation, invasion, angiogenesis and resistance to apoptosis, thus contributing to more aggressive diseases such as cancer [23]. However, the understanding of how integrin ligands could activate or inhibit this cross-talking is far from being understood. 

Binding of ligands to integrins activates outside-in signaling, which triggers a vast array of intracellular signaling events that determine cell fate, as above mentioned. As such, tight regulation of signaling via integrins is paramount for normal physiological function, and misregulated integrin activity is associated with many pathological conditions including cancer.

Many studies were devoted to find integrin antagonists, such as antibodies, peptides, and small organic molecules, which inhibit integrin function. Preclinical studies suggested that antagonists of several integrins might be useful for suppressing tumor angiogenesis and growth either alone, in combination or by conjugation with current cancer therapeutics [5,24]. 

Less attention focused on ligands that activate integrins instead of inhibiting them for possible activation of angiogenesis and tumor growth. However, it was recently recognized that integrin agonists could open novel opportunities for therapeutics, which have benefits in increasing rather than decreasing integrin-dependent adhesion. For instance, a significant factor in chemo-resistance in melanoma is a loss of integrin-mediated adhesion; in this case, stimulation of integrin signaling by agonists significantly improved the response to chemotherapy [25].

In this paper, we overviewed the literature about small molecules that directly target integrins and revealed an activating action on some of them, especially those involved in cancer. These agonist molecules were examined in terms of structure and behavior from a medicinal chemistry point of view. 

## 2. Agonists or Antagonists: That Is the Question

The complex roles of integrins in several pathologies identify this family of adhesion receptors as valuable drug targets. To date, most efforts have prompted the development of small molecules targeting integrins, especially those implicated in cancer (such as α_v_β_3_, α_v_β_5_, α_5_β_1_), in platelet aggregation (α_II_β_3_), and in the regulation of inflammation and immune functions (such as α_4_β_1_, α_4_β_7_, α_L_β_2_ and α_M_β_2_), although to date, no anticancer drug targeting integrins has been approved.

As previously described, integrins can mediate their own functions by changing conformation, as they exist in a dynamic equilibrium of several conformations that are determined by ligand interaction, or that induce ligand binding (Figure 2). As classical signaling receptors, following ligand binding, integrins promote signal transduction processes. Since several reports have shown that different ligands behave in a dissimilar way, it has been hypothesized that integrin ligand binding could mediate more than one downstream signaling mechanism [26]. On the basis of this hypothesis, the binding of different ligands may result in distinct events.

Pharmacologically, ligands can be classified on the basis of their action at the receptor. Agonists are compounds which bind to receptors and mimic the signaling of endogenous compounds; on the contrary, antagonists bind to the receptor and block its interaction with endogenous agonists, but do not induce any receptor activation and signal transduction, and therefore do not possess intrinsic activity. Additionally, some ligands, defined as partial agonists, possess less ability to activate the receptor and associated signal transduction, while inverse agonists are compounds that are able to stabilize the receptor in its inactive conformation. Moreover, a ligand can modulate receptor activity or binding of agonists/antagonists acting allosterically on a topographically distinct position from the site of activity or ligand binding: they are defined as allosteric agonists/antagonists.

All these definitions may apply to integrins and integrin-mediated signal transduction, suggesting that it could be possible to develop small molecules to target specifically a definite integrin conformation and downstream signaling: a sort of “biased ligand” for integrins [27]. Several studies suggest that integrins may share this feature with G-protein coupled receptors (GPCRs) for which “biased agonism” or differential signaling has been a deeply studied phenomenon over the last 10 years [28]. However, further investigations will be necessary to deepen our knowledge on these processes in integrin functions.

Recently, several studies have reported the development of small molecules acting as integrin agonists, that may display potential clinical applications. Analyzing those ligands, it has been recognized that not all agonists behave in the same manner, supporting the idea of biased agonism for integrins.

**Figure 3:**
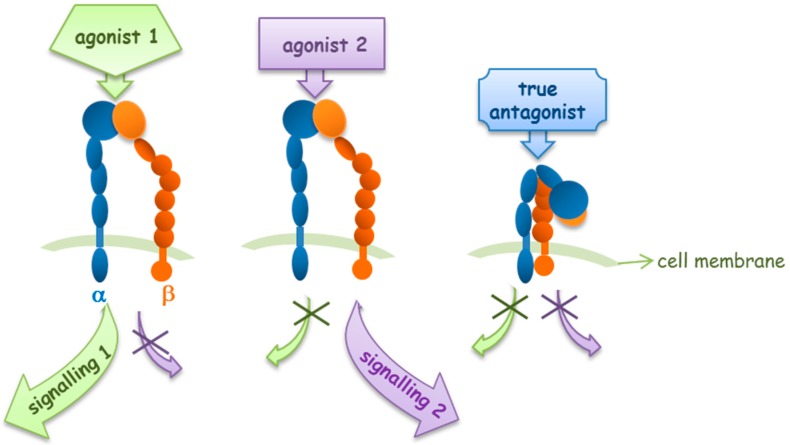
Integrin agonists and antagonists behavior in outside-in signaling pathways.

Integrin activation and signal transduction may be differently modulated by different integrin agonists (Figure 3). Faridi and coworkers reported that small molecule agonists for the α_M_β_2_ integrin induce modest and local changes in integrin structure, while the binding of activating antibodies, which activate integrins or that bind in an activation-sensitive manner, prompt more global conformational changes, inducing outside-in signaling [29].

These new insights into integrin functions require a better characterization of synthetic compounds (both agonists and antagonists): it is crucial to investigate deeply their effects on several functions mediated by integrins such as cell adhesion, intracellular signaling and integrin trafficking. Furthermore, integrin internalization regulates several processes, like cell migration and adhesion, and is relevant in many pathological conditions, especially in cancer [30,31]. It would be interesting also to investigate the effects of integrin agonists and antagonists also on integrin trafficking: for example, these data could be useful to develop small molecules able to deliver selectively cytotoxic molecules into cancer cells. It is currently unclear whether integrin agonists and antagonists may mediate internalization in a different way. 

## 3. RGD-Binding Integrin Agonists

**Figure 4:**
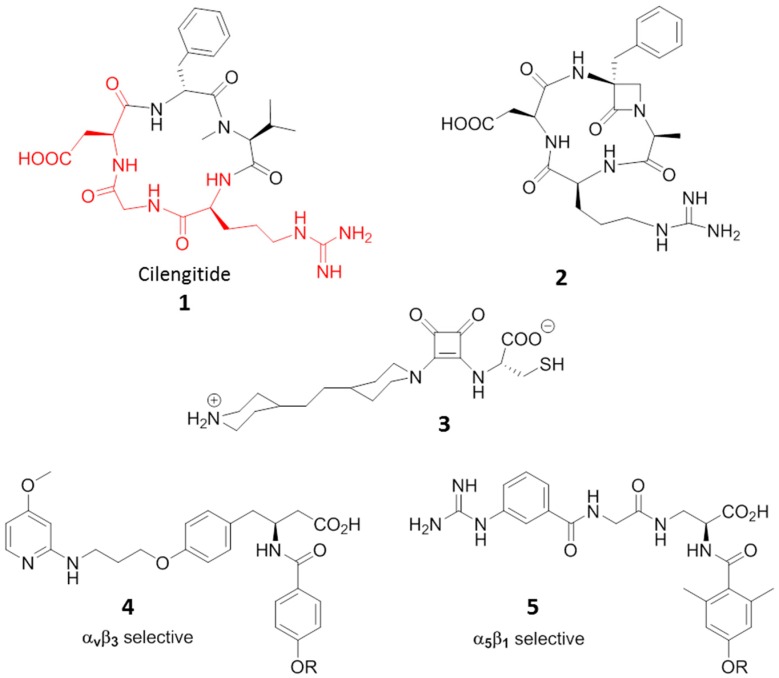
Ligands to RGD-binding integrins displaying agonist behaviour. RGD sequence in Cilengitide is highlighted in red.

Cilengitide, the cyclic RGD pentapeptide c(-RGDf(NMe)V-), is a potent integrin antagonist targeting the integrins α_v_β_3_, α_v_β_5_ and α_5_β_1_, developed for treatment of glioblastomas and other tumors (Compound **1**, Figure 4) [32]. Its features and potency boosted research in the last two decades towards a plethora of new small molecules able to antagonize integrins [33,34,35]. The inhibition of integrin-ligand interactions suppresses cellular growth and induces apoptotic cell death, and in this context, Cilengitide has been reported to be a potent inhibitor of angiogenesis able to induce apoptosis of growing endothelial cells via the inhibition of the interaction between integrins with their ECM ligands.

Despite great expectations, antagonists of α_v_β_3_ and α_v_β_5_ integrin that have entered clinical trials as antiangiogenic agents for cancer treatment have generally been unsuccessful. In phase III studies, the addition of Cilengitide to temozolomide chemo-radiotherapy failed to be effective and therefore, Cilengitide will not be further developed as an anticancer drug. Nevertheless, integrins remain a potential treatment target for glioblastomas [36].

The full concept of exploiting integrin antagonism as an antiangiogenic therapy was criticized in 2009, in a study [37] reporting that low-dose treatment with RGD-mimetic integrin antagonists may paradoxically enhance angiogenesis and tumor growth. This behavior has been ascribed to in vivo evidence that low (nanomolar) concentrations of RGD-mimetic α_v_β_3_ and α_v_β_5_ antagonists can paradoxically stimulate tumor growth and tumor angiogenesis. Yet in 2001, Legler and coworkers confirmed that cyclic RGD-peptide (cRGD) acted as an antagonist on α_v_β_3_ at high concentrations, and as agonist at low concentrations [38]. Even if the Cilengitide concentrations used in clinical trials far exceeded the described “pro-angiogenic” concentrations, and therefore the adverse biological effect should not be expected, these findings suggest that nanomolar plasma concentrations of those compounds with ambivalent behavior should be avoided, or that their delivery should be redesigned.

The ambivalent behavior of Cilengitide prompts the need for better understanding of how binding events modulate integrin activity, and the role of a ligand as an agonist or an antagonist with the perspective of designing new compounds that are unable to promote integrin activation, and thus can act as pure antagonists or vice versa as pure agonist for internalization studies and drug delivery. In a recent molecular dynamic (MD) study [39], authors analyzed the multidomain receptor of α_v_β_3_ integrin in complexes with two forms of fibronectin, wild type (wtFN10) and mutated high affinity (hFN10), which act, respectively, as an agonist activating the receptor, or as a true antagonist inhibiting the receptor. Interaction hotspots were identified in the integrin binding site that specifically respond to the fibronectin sequence variations and allosterically drive conformational changes towards integrin activation or inhibition. They speculated that antagonism is determined by the presence of bulky moieties, i.e., aromatic, flanking RGD that optimally pack in the integrin recognition site. Agonism is favored by the absence of such flanking motifs, which allows more conformational freedom and pushes integrin towards the active conformation.

Other molecules displaying agonist or ambivalent behavior towards RGD binding integrins have also been reported. Aizpurua and coworkers [40], designed and synthesized RGD cyclic peptidomimetics incorporating an α-amino-β-lactam scaffold. The antagonist affinities against α_v_β_3_ integrin on human endothelial cells (HUVECs) by means of adhesion inhibition assays resulted in comparable values to that of Cilengitide. On the contrary, gene expression microarray assays on angiogenesis-related gene regulation of DNA samples extracted from HUVECs after treatment with the RGD, in case of β-lactam ligand 2 (Figure 4), gave opposite behaviors with respect to Cilengitide, suggesting an in vivo proangiogenic effect by the compound which might act as an agonist ligand of the RGD receptor. 

Once again in the field of mimics of the natural RGD sequence, Luk [41] reported on a class of squaramide molecules that exhibited higher potency at inhibiting mammalian cell adhesion than RGD tripeptides in culture medium inhibition tests. But compound **3** (Figure 4), when immobilized on a bio-inert surface resistant to non-specific cell adhesion, facilitated a faster (1.4 times) and stronger focal adhesion than linear RGD ligands, suggesting that 3 could induce more adhesion points within an adhered cell, and thus should be considered as an agonist for integrin rather than antagonist. The ability to increase cell adhesion when immobilized on inert surfaces has also been demonstrated by some RDG peptides [42] and by small molecules. Mas-Moruno [43] studied the immobilization of integrin-binding peptidomimetics (compounds **4** and **5**, Figure 4) on titanium (Ti) as a feasible and powerful strategy to mimic a bone extracellular matrix, and thus to improve osteoblast adhesion and accelerate osseointegration of implants. Compounds **4** and **5** are α_v_β_3_ and α_5_β_1_ selective agonists, respectively, and both fostered adhesion and spreading of SaOS-2 cells on Ti, thus opening promising prospects for diverse clinical applications of agonists in dentistry and orthopedics.

**Figure 5:**
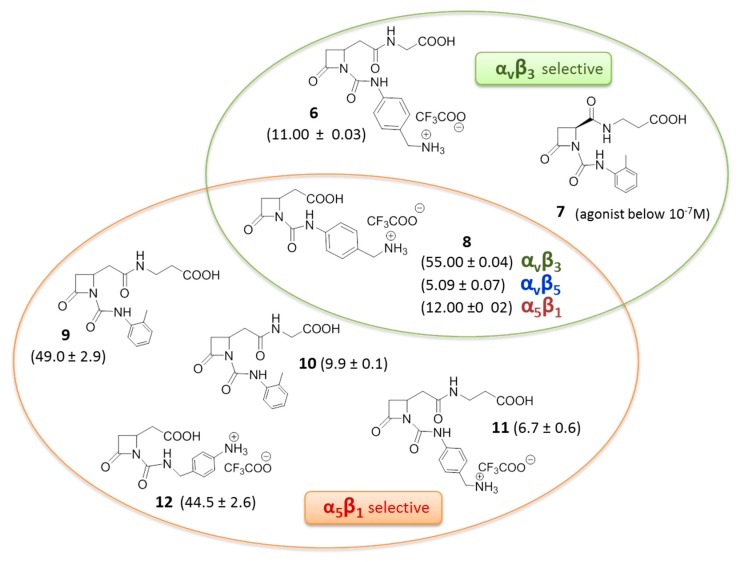
β-Lactam-based agonists to RGD-binding integrins, in parenthesis half maximal effective concentration EC_50_ (mean ± SD, nM).

Recently, a novel series of β-lactam derivatives designed and synthesized to target RGD-binding and leukocyte integrins, was reported (Figure 5) [44,45]. The molecules contain an azetidinone ring as a rigid cyclic central core, with two arms holding a carboxylic acid and a basic moiety, as in the RGD sequence, or a carboxylic acid side chain coupled with a 4([(*N*-2-methylphenyl)ureido]-phenyl acetyl motif (PUPA) [46] on the β-lactam nitrogen atom. These new ligands were evaluated by investigating the effects on integrin-mediated cell adhesion and cell signaling in cell lines expressing α_v_β_3_, α_v_β_5_, α_v_β_6_, α_5_β_1_, α_IIb_β_3_, α_4_β_1_, and α_L_β_2_ integrins, and in solid phase binding assays. Interestingly, several of these derivatives were found to be good antagonists, and by modulating the basicity and the length of the side chains, selective and potent agonists were also found. In particular, several compounds could induce cell adhesion and promote cell signaling mediated by α_v_β_3_, α_v_β_5_, and α_5_β_1_ integrins. The more potent β-lactam-based agonists are depicted in Figure 5, where the selected molecules showed EC_50_ values ranging from 1 to 100 nM. In particular, β-lactams 8–12 showed higher and sometimes selective affinity toward α_5_β_1_ integrin, whereas β-lactam 6 and 8 showed affinity toward integrin α_v_β_3_. Compound **7** with an acidic terminus of β-alanine and an ureido PUPA motif behaved as an antagonist toward the α_v_β_3_ integrin at low concentrations, and as an agonist at higher concentrations, thus mimicking the concentration-dependent behavior of Cilengitide, but with an opposite trend. It was demonstrated that cell adhesion mediated by the new β-lactam agonists effectively and specifically involved α_5_β_1_, α_v_β_3_, and α_4_β_1_ integrins, respectively. Regarding α_v_β_3_ integrin, adhesion of melanoma cells was increased in a concentration related-manner by compounds **6** and **8**, as well as by fibronectin. Moreover, pre-incubation with a cyclic RGD mimetic c(-RGDfV-), a well-known antagonist of RGD integrins, significantly reduced melanoma cell adhesion mediated by compounds **6** and **8**, thus suggesting that both β-lactam molecules may bind to the MIDAS site as c(-RGDfV-). The same experiment on α_5_β_1_ expressing cells, showed reduction of cell adhesion mediated by azetidinones 9 and 12 but not by 10 and 11, suggesting that β-lactams 9 and 12 may bind the MIDAS site, whereas 10 and 11 might bind to an allosteric site. Pre-incubation with a neutralizing antibody against the α_5_ or α_v_ subunits blocked the augmented adhesion induced by all β-lactam agonists 6 and 8–12, thus inducing the hypothesis that compounds **10** and **11** could bind to allosteric sites specifically located on α subunits.

Starting from the hypothesis that a loss of integrin-mediated adhesion is a significant causative factor in chemo-resistance in melanoma cells, it has been shown that integrin agonists can act as adjuvants in chemotherapy [25]. Disintegrins are a group of integrin-binding proteins found in snake venoms. Contortrostatin is a disintegrin possessing two RGD motifs that, on binding to α_v_ and α_5_β_1_ integrins usually present on cell surface of melanoma cells, is able to inhibit cell adhesion and platelet aggregation, but to activate integrin-mediated signaling. In an in vivo model, the combined treatment of contortrostatin with araC (Cytarabine, a chemotherapy drug) significantly decreased tumor growth, probably due to the ability of contortrostatin to stimulate integrin-mediated signaling, leading to a significantly improved response to chemotherapy. These data suggest that combining chemotherapy with integrin agonists may be promising for improving therapeutic outcomes in patients with metastatic melanoma [25].

**Figure 6:**
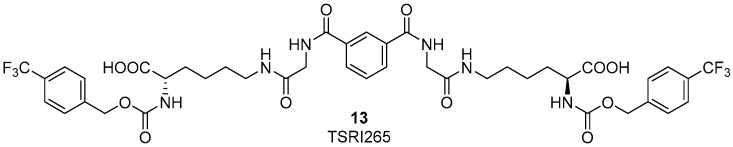
Chemical structure of TSRI265, an α_v_β_3_ ligand able to disrupt the integrin-MMP2 interaction and showing antiangiogenic activity.

Several years ago it was discovered that integrins can interact and colocalize with MMP2 on the surface of angiogenic blood vessels in vivo [47]. The binding of α_v_β_3_ integrin to MMP2 on the cell surface is fundamental for the exploitation of this enzyme on the cell surface of invasive endothelial cells. Therefore, the development of new ligands, binding to α_v_β_3_ integrin and as a consequence disrupting MMP2-α_v_β_3_ integrin interaction would be interesting for anti-angiogenic therapy in cancer. Silletti et al. reported some integrin ligands [48,49] which did not bind to the RGD binding site of α_v_β_3_ integrin, did not alter MMP2 catalytic activity directly, and did not interfere with the binding of α_v_β_3_ integrin to the ECM endogenous ligand vitronectin. Moreover, these compounds inhibited tumor cell capacity to use MMP2 to degrade ECM by disrupting the integrin-MMP2 protein interaction. In in vivo models, the best candidate, compound **13** (TSRI265, Figure 6) showed potent antiangiogenic activity and inhibited tumor growth [49].

## 4. Leukocyte Integrins

The mutual relationship between inflammation and cancer is nowadays well established, and immunomodulation is considered a useful tool not only for the treatment of inflammatory pathologies and autoimmune diseases, but also in cancer therapy [50,51]. When the release of chemokines and growth factors due to inflammation is chronic, oxidative damage and DNA mutations may occur, thus supporting tumor development [52]. On the other hand, the effects of cancer on the immune system include several pathways such as the up-regulation of immune-suppressive cytokines, and the dysregulation of T-cell mediated host responses. In this context, the role of adhesion molecules is fundamental for leukocyte recruitment and migration and for T-cell infiltration in tumor tissues. Moreover, regulation of the expression and activity of specific adhesion molecules has a strong impact on B-cell homing, survival and environment mediated drug resistance in malignancies such as non Hodgkins lymphoma (B-NHL) [53,54]. An important aspect is the binding-detachment ratio that controls cell movement and is responsible for the dynamic aspects of the process. Recent studies have shown that fast leukocyte recruitment to the site of injury occurs during acute inflammation, while slower processes may be observed for the immune response in pancreatic and hepatic cancer [55]. In this complex scenario, modulation of integrin activity plays a fundamental role. While antagonists may interfere in leukocyte primary functions, agonists may induce a stronger adhesion that, avoiding detachment, prevents normal cell migration processes [56]. Leukocytes express on their surface, selected classes of integrins (see Figure 1), and, among them, α_L_β_2_ (LFA-1, CD11a/CD18), α_M_β_2_ (CD11b/CD18, Mac-1), α_4_β_7_ (LPAM) and α_4_β_1_ (VLA-4) received major attention as targets for small molecule ligand-induced immunomodulation.

### 4.1. β_2_ Integrin Agonists

Like other integrins, those belonging to the β_2_-family possess the αI-domain as the main ligand-binding site in the α subunit, as mentioned above. This domain is a 190–200 residue fragment at the N-terminus of the α chain, with seven α helices surrounding a mostly parallel β sheet, which contains a MIDAS. A similar domain, named the I-like domain, is present also in the β_2_ subunit. The crystal structure of α_L_ (CD11a) and α_M_ (CD11b) I domains allowed two different conformations to be identified [57,58]: a low-affinity quaternary state (closed-inactive) and a high-affinity state (open-active). When ligand-induced activation occurs, a β_2_ I-like domain rearrangement activates the αI domain [59]. Reported antagonists usually stabilize the low-affinity confirmation of I domain and allosterically inhibit ligand binding (αI allosteric antagonists). Other known antagonists bind to the I-like domain in the α-unit, activating this portion of the receptor but blocking the activation of the α-unit (α/β I allosteric antagonists) [57].

**Figure 7:**
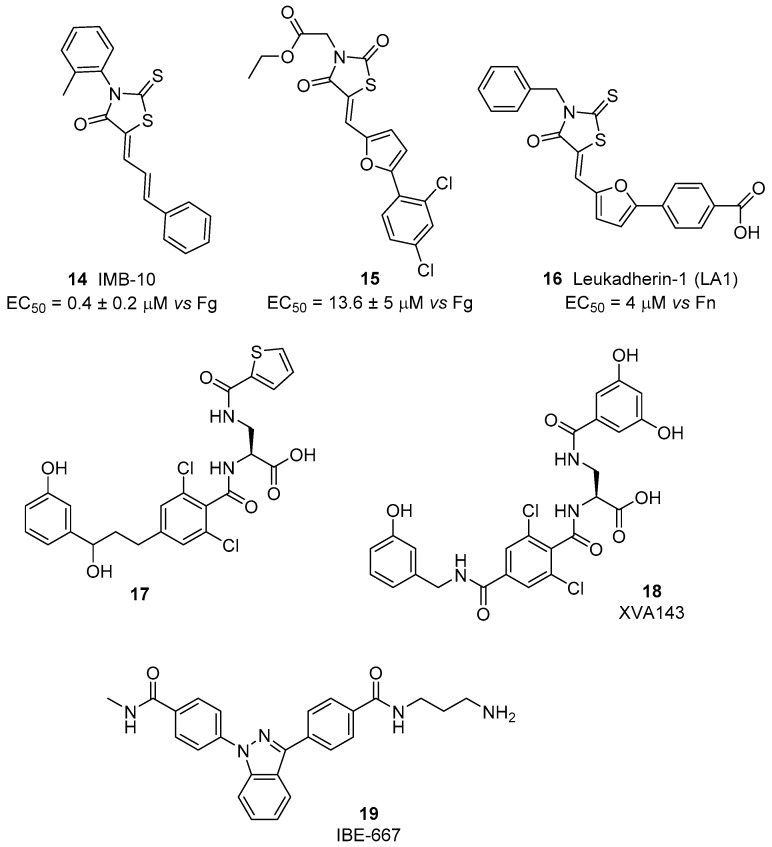
β_2_ integrin agonists; data are expressed as EC_50_ (mean±SD, nM) vs. ligand employed in cell adhesion assay.

The idea that activation instead of inhibition of leukocyte integrins may represent a useful approach for inflammatory disease treatment is supported by the finding that induction of a persistent active state may lead to the loss of integrin mediated functionality [60]. Initially, small molecule agonists of β_2_ integrins have been identified starting from the DDGW peptide that mimics the binding sequence of matrix metalloproteinases (proMMP)-2 and -9 to these receptors. Via a high-throughput phage display screening, a small library of 2-thioxothiazolidin-4-one derivatives was selected for their specific binding affinity to the α_M_ and α_L_ I domains. Compound **14**, IMB-10 (Figure 7), inhibited competitive antibody binding and showed an interesting ability to increase binding of α_M_β_2_ integrin to matrix metalloproteinases and fibrinogen (EC_50_ = 0.4 ± 0.2 μM) [61]. Docking studies suggested the presence of a hydrophobic cavity able to host IMB-10 close to the C-terminal helix in the open form of the α_M_ subunit. In the closed inactive form, this cavity is not available, since it is occupied by some residues of the C-terminal helix. The phenylbutadienyl chain of IMB-10 fits to the bottom of the pocket, while the 2-methylphenyl group establishes aromatic stacking with a phenylalanine residue of the receptor. A further hydrogen bond occurs between the carbonyl group of the heterocyclic core and a serine hydroxyl function. A similar pocket is present also in the α_L_ subunit, but the calculated fitting was less effective. This interaction between IMB-10 and the α_M_ I domain strongly stabilizes the open active form of the receptor, preventing the switch back to the closed inactive form, where the pocket needs to be empty to allocate the C-terminal helix. Additional experiments also showed that IMB-10 is able to block α_M_β_2_-mediated cell migration in vitro and leukocyte recruitment in vivo; thus, confirming the potential of this small molecule as an anti-inflammatory lead compound. Furthermore, this compound displayed anticancer activity in in vivo models [56]. Interestingly, IMB-10 reduced leukocyte infiltration in tumors and altered the invasion ability of cancer cells, probably blocking integrin-mediated inflammatory cell recruitment. Therefore, IMB-10 could be a promising lead for the development of therapies to fight leukocyte-originating malignancies.

A screening of >13,500 compounds via a no-wash cell adhesion-based high throughput assay [62], allowed the identification of a novel family of compounds able to increase adhesion to the fibrinogen of human erythroleukemia cells, transfected with α_M_β_2_ integrin. The common motif of these compounds’ backbone is the presence of the 2-thioxothiazolidin-4-one heterocyclic core, as in IMB-10. By merging information obtained from structure-activity relationship study and in-silico docking experiments, the interaction of these agonists with the hydrophobic pocket between the α_7_ and α_1_ helixes of I domain was confirmed [63]. In particular, compound **15** (Figure 7), possessing a planar conjugated furanyl aromatic chain, best fits into the cavity, orienting the carbonyl oxygen toward serine and threonine residues able to establish stabilizing hydrogen bonds. Further development of this class of compounds, named leukadherins, allowed the identification of three ligands with excellent activity in vitro (Leukhaderin-1 LA1, Leukhaderin-2 LA2 and Leukhaderin-3 LA3, compounds **14**–**16**, Figure 7) [64]. Among them, LA-1 (compound **16**) was selected as the lead compound, increasing the α_M_β_2_ integrin mediated adhesion to fibrinogen with an EC_50_ value of 4 μM. Further experiments confirmed that LA-1 binds to the αI domain in the high affinity conformation through allosteric stabilization of the αI in the open active form. Moreover, leukadherins reduce leukocyte trans-endothelial migration in vitro and decrease recruitment and extravasation in vivo. However, they did not behave as ligand mimics, since receptor clustering and outside-in signaling did not occur upon binding to the receptors. Comparison of the function of leukhaderins as agonists to antibodies suggests that these small molecule ligands are able to induce conformational changes limited to the αI domain, which is not sufficient to activate intracellular signaling via the complex cytoskeleton protein machinery. On the contrary, binding of agonist antibodies mimics the natural ligand and induces extension of the heterodimer with a global conformational rearrangement. This activation produces outside-in signaling, but sometimes leads to adverse effects [29]. An insight on LA-1 binding effects was performed with atomic force microscopy (AFM)-based single-cell force spectroscopy (SCFS), by comparing the biomechanical effects of leukadherin-specific ligands with those induced by Mn^2+^, a specific integrin agonist [65]. These investigations revealed the existence of two distinct populations of receptors: one with a strong linkage to the cytoskeleton and which is activated by interaction with Mn^2+^, the other with weaker connections that are broken when leukadherin binding occurs, inducing formation of membrane tether bonds. Thus, LA-1 binding leads to conversion of the closed inactive form of the receptor to an intermediate affinity conformation, different from the open-active form induced by other classes of agonists (Figure 2). This novel mode of action did not show significant side effects so far, thus prompting novel in vivo studies on inflammatory disease animal models [66,67].

A different mechanism of activation occurs for compound **17** (Figure 7) [68], a small molecule sharing structural similarities with α/β I allosteric antagonists studied by Genentech and Hoffmann-Laroche [69]. This α_L_β_2_ ligand displayed agonist activity in the presence of physiologic divalent cations as Ca^2+^ and Mg^2+^, but behaved as an adhesion inhibitor in the presence of Mn^2+^. This unusual behavior was confirmed in several different assays as binding tests, static cell adhesion and flow chamber assays, both with human erythroleukemia cells transfectants expressing α_L_β_2_ and physiologic leukocytes. The proposed hypothesis is that cations may bind to an adjacent MIDAS (ADMIDAS), generating complexes that are slightly different depending on the metal. For Ca^2+^ and Mg^2+^, the complex between compound **17** and the β I-like domain can bind to α_L_ I domain, inducing a switch to the open active conformation. Recently, XVA143 (compound **18**, Figure 7), a α/β I allosteric antagonist, having structural similarities to compound **17**, was reported to induce a semi-active form of α_L_β_2._ This conformation induces “rolling adhesion” as the beginning of leucocytes recruitment, but does not mediate intracellular signaling and promotes an altered internalization/recycling behavior. The splitting of different down-stream effects depending on the binding site and on epitope formation suggests that much is still to be understood [70].

Based on SAR information obtained from on-bead screens of tagged one-bead one-compound combinatorial libraries, a novel α_L_β_2_ ligand acting as agonist was identified [71]. The small molecule 19, named IBE-667 (Figure 7), increased the binding of biotinylated soluble ICAM-1 to activated T-cells, thus acting as an ICAM-1 binding enhancer for LFA-1. Co-crystallization experiments revealed that IBE-667 does not bind to the MIDAS site of LFA-1 but instead to a pocket usually preferred by allosteric antagonists such as Lovastatin (α_L_ domain).

### 4.2. α_4_β_1_ Integrin Agonists

The α_4_β_1_ integrin is a key player in the homing of progenitor cells to inflammation sites and mediates cell adhesion to VCAM-1 and to the alternatively spliced segment-1 (CS1) of fibronectin. This receptor lacks an I domain, and ligand binding occurs at the interface between α and β subunits, through coordination to a MIDAS in the α subunit. Crystal structure of α_4_β_1_ integrin is still not available, and all the designed ligands have been planned based on the structure of α_4_β_7_ integrin that shares the same α subunit [72].

**Figure 8:**
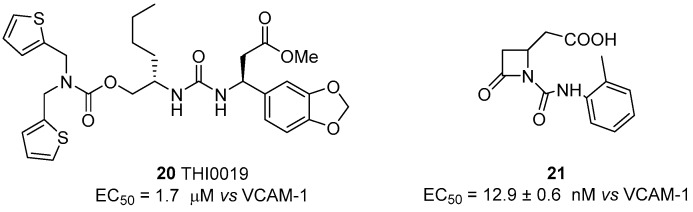
α_4_β_1_ integrin agonists; data are expressed as EC_50_ (mean±SD, nM) vs. ligand employed in cell adhesion assay.

As reported above [60], potential application of integrin agonists as therapeutic agents started after the observation that freezing the α_4_β_1_ integrin (VLA-4) in a high-avidity state by activation with monoclonal antibodies generated a strong interference with trans-endothelial migration of leukocytes. Although this study suggested a novel approach to prevent rapid tissue invasion by VLA-4-positive cells during inflammation, α_4_β_1_ integrin agonists have been poorly explored in comparison with antagonists. The first agonist THI0019 (compound **20**, Figure 8) was synthesized using a potent antagonist as template, by introducing a methyl ester instead of a carboxylic function, thus avoiding MIDAS affinity [73]. Cell adhesion assays with T lymphocyte cells in the presence of VCAM-1 showed an enhancement of cell binding with an EC_50_ of 1.2 μM; comparable agonist activity was also observed toward α_4_β_7_, α_5_β_1_ and α_L_β_2_ integrins, while negligible effects were observed on α_v_β_3_, α_1_β_1_ and α_2_β_1_ integrins. Further bioassays, supported by docking experiments based on the α_4_β_7_ crystal structure, suggested that THI0019 temporarily occupies the ligand binding pocket, bridging both α and β subunits and inducing a small conformational change in the β unit that favors ligand binding and agonist displacement, in agreement with the binding mode of other small-molecule integrin systems [72,74]. Different from previously described β_2_ integrin agonists, THI0019 is a full agonist and promotes rolling, migration and cell homing, and, in this case, the up-regulation of integrin activity should be useful for cell retention in stem cell therapy. In fact, in patients who have had a myocardial infarction, early retention of transplanted stem cells may significantly improve heart functions [75,76]. Co-administration of stem cells with a α_4_β_1_ integrin agonist such as THI0019 may potentially improve cell retention at the site of injury, and in this way enhance stem cell therapy.

The switch from antagonist to agonist behaviour, due to small changes in the molecule backbone, was also recently reported for β-lactam ligands, designed to target RGD-binding and leukocyte integrins [45]. A library of small molecules was indeed synthesized and screened for the affinity and selectivity to different classes of integrins. Within this study, compound **21** (Figure 8) was found to be a selective agonist of α_4_β_1_ integrin, having an EC_50_ of 12.9 nM in T lymphocyte cell adhesion assays to VCAM-1, and activating outside-in signaling, evaluated quantifying ERK1/2 phosphorylation. The binding mode of the lactam was further explored by using a conformation-specific anti-β1 integrin antibody (HUTS-21) able to recognize a ligand-induced binding site (LIBS) epitope that is exposed upon agonist binding or partial integrin activation. Agonist 21 significantly increased HUTS-21 antibody binding in a concentration-dependent manner, thus demonstrating the induction of a conformational rearrangement in the β_1_ subunit that resulted in exposure of the HUTS-21 epitope and a more active conformation.

## 5. Laminin-Binding Integrins

Engagement of laminins by a group of integrin family proteins, including integrins α_3_β_1_, α_6_β_1_, α_7_β_1_ and α_6_β_4_, is an important event in the interaction of cells with basement membranes. Different isoforms of both integrins and laminins showed specific affinities in binding studies, thus inducing different effects in cell physiology [77]. Laminin-binding integrins have been suggested to be potent mediators of tumor cell motility, migration and invasion during metastasis but, depending on the conditions, they may have pro-metastatic or anti-metastatic functions [78].

Within this class of receptors, integrin α_3_β_1_ [79] is highly expressed in podocytes, terminally differentiated visceral epithelial cells, and primarily binds to laminin expressed in the glomerular basement membrane (GBM). Integrin α_3_β_1_ mediates stable adhesion and maintains the integrity of podocytes and the glomerular filtration barrier. Damages or protein mutations that reduce either integrin activation or expression, may result in podocyte alteration and may cause proteinuria. 

**Figure 9:**
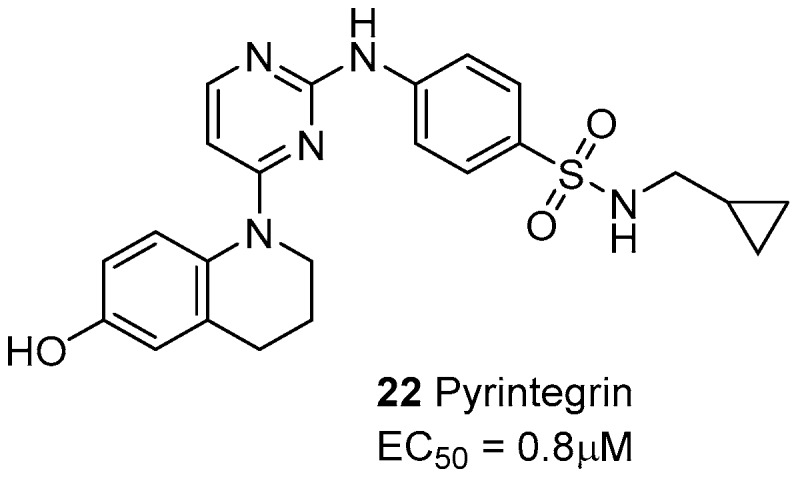
Laminin-binding integrin agonists.

The small molecule pyrintegrin (Figure 9) behaves as an activating/protecting agent of integrins from puromycin aminonucleoside (PAN)-induced damage, via a dose-dependent effect on the podocytes (EC50 = 0.8 μM) [80]. Moreover, pyrintegrin showed a strong survival-promoting effect on dissociated human embryotic stem cells (hESCs), inducing in a few hours a dramatic increase in the adhesion to laminin-coated plates. Further experiments confirmed that pyrintegrin increases integrin activity and activates intracellular signaling [81]. This molecule was also capable of promoting adipose tissue formation from either transplanted human adipose stem/progenitor cells or host endogenous cells, both in vitro and in vivo. The efficacy in endogenous adipogenesis without cell transplantation suggest that pyrintegrin treatment may be also exploited as an alternative to autologous fat transfer in post-operative volume reduction [82].

## 6. Collagen-Binding Integrins

Under disease conditions, such as inflammation, tissue regeneration events, and tumors, collagen-binding integrins α_1_β_1_, α_2_β_1_, α_10_β_1,_ and α_11_β_1_ play a more-prominent role. Integrins α_1_β_1_ and α_2_β_1_ might be needed for a correct and innate immune response in various populations of immune cells, whereas α_10_β_1_ and α_11_β_1_ appear to be central to how connective tissue cells perform in the musculoskeletal system [83].

Examples of small molecules that act as agonists of collagen-binding integrins are very poor. Chung et al. reported on the effect of some snake venom toxins such as the C-type lectin protein Aggretin, which induces vascular smooth muscle cell proliferation and migration, and stimulates the signaling pathways via activation of α_2_β_1_ [84]. However, a recent contribution by the same authors reported that a small mass peptide fragment of aggretin may bind integrin α_2_β_1_ and acts as antagonists of angiogenesis, thus reversing the behavior compared to aggretin [85].

## 7. Conclusions

Integrins are crucial transmembrane receptors, whose action mode is quite complex and not yet completely understood. Integrins interconnect extracellular and intracellular compartments, thus acting as a bidirectional hub transmitting outside-in or inside-out cellular signals.

It was ascertained that activation of integrins occurs via conformational rearrangement from bent-closed and open-active states in the extracellular domain. Activation state of integrins depends on binding to specific ligands, and it is important to point out that the strength of this binding (affinity) could be modulated by various factors, including the status of glycosylation of integrin, as recently reported [21,22].

Less attention has been devoted to agonist ligands that activate integrins. Integrin agonists could open novel opportunities for therapeutics, which gain benefits to increase rather than decrease integrin activities, such as adhesion. As an example, it was reported that the activation of integrin β1 attenuates invasion by stabilizing adhesions high integrin activation in a 3D matrix [86].

Although the potential therapeutic application of integrin agonists exists, it is important to ascertain the exact behavior of new integrin ligands (antagonist or agonist) towards several cellular events mediated by integrins, such as adhesion, signaling, clustering, and trafficking. In case of an ambivalent role (antagonist and agonist together) it would be extremely important to determine if this is concentration-dependent, such as the case of Cilengitide. Appropriate knowledge of this aspect should limit possible failures at a late stage of clinical trials of future lead compound.

Given the key role exploited by integrins in cancer, it would be important to deepen our knowledge in this field. In particular, integrin agonists have to date been studied only as chemotherapy adjuvants in melanoma tumor [25], therefore, it would be useful to better understand which therapeutic role integrin agonists could have in cancer, and in different tumor models, but extreme caution must be taken after the case of Cilengitide.

Moreover, it is important to establish if an integrin agonist behaves as an allosteric ligand. An allosteric ligand could have the advantage of the ability to prime the integrin on leaving the main binding site free, thus allowing processes such as integrin clustering or other interactions with cytokines to occur, as reported for allosteric agonist antibodies of β_1_ integrins, which activate cytokine TGF-β in melanoma. This mechanism results in stromal activation, neo-angiogenesis and an increase in the number of T lymphocytes within the tumor microenvironment, which attentuated tumor growth and conferred long-term survival benefit [87].

Another important point to be addressed is the effect of ligands (agonists and antagonists) on integrin trafficking and internalization, because these processes regulate several events such as cell migration and adhesion, and are relevant in many pathological conditions, especially in cancer. Investigations on integrin internalization could be useful to exploit small molecules such as drug cargoes, which able to selectively bind and activate integrins, and to deliver cytotoxic molecules into cancer cells via integrin endocytosis. Now, it is currently unclear whether integrin agonists and antagonists mediate internalization in a different way. 

The realization that different ligands can activate or inhibit integrins in a dissimilar way and that “biased agonism” could be applied also to integrins, suggests that it should be possible to design synthetic agents that specifically target an integrin-mediated effect. Therefore, a better and complete characterization of small molecules that behave as integrin agonists or antagonists will be paramount for the development of novel drugs that target integrins with high potential and reduced adverse effects.
